# Carbon emissions from total intravenous vs. volatile anaesthesia for elective cholecystectomy: a pilot observational study

**DOI:** 10.1111/anae.70167

**Published:** 2026-02-20

**Authors:** Aditya Krishnan, Stephen Berry, Katie Booth, Louise Hiller, Joyce Yeung

**Affiliations:** ^1^ University of Warwick Warwick UK; ^2^ Nottingham University Hospitals NHS Trust Nottingham UK

Every anaesthetic leaves an ecological footprint. Volatile anaesthetic agents are short‐lived climate pollutants with high global warming potential, with total intravenous anaesthesia (TIVA) considered a ‘greener’ alternative. Yet, most comparisons of anaesthetic techniques rely on modelling or retrospective records, which are unable to capture item‐level, real‐time practice [1]. Therefore, this pilot study assessed the feasibility of a prospective observational approach using pre‐existing life cycle assessment data to develop a life cycle emissions analysis for surgeries employing either anaesthetic technique, spanning anaesthetic, surgical and recovery periods [2].

We recorded prospectively every drug and item of equipment used in 16 consecutive day‐case laparoscopic cholecystectomies in adult patients (age > 18 y) at a UK centre in 2024, documenting actions in real time in the anaesthetic room, operating theatre and recovery, alongside gas flows from the anaesthetic machine. Carbon emissions were calculated for each surgery using existing process‐based life cycle assessments and environmentally extended input–output methods, expressed as carbon dioxide‐equivalents (kgCO_2_e) over a 100‐year global warming horizon and cradle‐to‐grave boundary where available [3]. Statistical analysis was descriptive and this study was not powered to detect differences between techniques.

Nine cases employed TIVA and seven used volatile anaesthesia. We calculated life cycle emission analysis across the 169 unique drugs and equipment (online Supporting Information Table S1). Mean (SD) total emissions per case were 45.2 (17.5) kgCO_2_e, of which anaesthetic‐related items contributed approximately one‐third, with surgical consumables dominating the overall footprint. Although underpowered, there was no significant difference detected between total emissions per case with the use of TIVA compared with volatile anaesthesia (mean (SD) 39.6 (5.5) kgCO_2_e vs. 52.4 (24.8) kgCO_2_e, respectively; p = 0.142). Nitrous oxide, utilised in two cases involving volatile anaesthesia, was the disproportionately single largest contributor (mean (SD) 28.3 (11.4) kgCO_2_e). Oxygen and air use was greater with TIVA (mean (SD) 472 (190) l vs. 264 (127) l, p = 0.023 and 166 (170) l vs. 33 (15) l, p = 0.008, respectively) reflecting the common practice of maintaining higher flows during TIVA.

Quantitative observations from this project are in keeping with previous studies, including the carbon dioxide emission equivalents from an elective cholecystectomy, the proportion of total emissions contributed to by anaesthetic drugs and equipment, and the gross carbon footprint of nitrous oxide [1]. This pilot study shows that prospective, granular, item‐level observation of a patient journey is achievable across anaesthetic, surgical and recovery areas. These data can be mapped to pre‐existing process‐based life cycle assessments and environmentally extended input–output data, without a new analysis, to produce life cycle emissions analysis to compare anaesthetic techniques. This approach allows comprehensive coverage of drugs and equipment while acknowledging inherent trade‐offs. Process‐based life cycle assessments offer specificity but may miss upstream and downstream processes outside the ecological boundary and environmentally extended input–output methods use cost as a proxy for resource intensity, which often fails to reflect its true ecological impact. In this study, the combination of both allows for hypothesis‐generation and planning prospective sample size calculations in future studies.

Several limitations constrain interpretation of this study, including: small sample size; exclusion of major emission sources (heating, ventilation, air‐conditioning energy and carbon dioxide used for abdominal insufflation); reliance on secondary life cycle assessment data with potential truncation error and varied system boundaries; and omission of complex products where proprietary composition prevented environmentally extended input–output methods estimation (e.g. Floseal haemostatic matrix) [4]. These limitations preclude conclusions about differences in emissions between techniques and explain incongruence with previous larger studies, which have relied predominantly on retrospective record analysis or modelling.

Current carbon accounting relies on the CO_2_e metric, which misrepresents short‐lived pollutants like volatile anaesthetic agents, which, unlike carbon dioxide, have brief atmospheric lifetimes [5]. Furthermore, this approach focuses solely on greenhouse gas‐related climate impacts, excluding environmental categories such as acidification; eutrophication; ozone depletion; and water use. Propofol that has been disposed of improperly (and associated metabolites) has been detected in water bodies [6], while volatile degradation products persist as ‘forever chemicals’. Nevertheless, CO_2_e remains the globally recognised currency for ecological impact and underpins international climate targets including the NHS Net Zero strategy, providing a common framework for comparison. Broader environmental assessments are encouraged to capture these additional impacts, which are poorly studied.

The value of this work lies in its methodological proof‐of‐concept and observational value. Future research should apply this approach to prospectively powered observational studies, enabling robust comparisons not only between TIVA and volatile anaesthesia but also across other techniques, such as regional anaesthesia with sedation vs. general anaesthesia. Further studies should broaden system boundaries, such as including heating, ventilation and air conditioning energy, which makes up over 90% of operating theatre electricity use. Establishing standardised carbon accounting in anaesthesia is crucial for advancing research into sustainable practice; this study marks another step toward that goal.

## Supporting information


**Table S1.** Summary of life cycle assessment methods used to determine CO_2_e.
