# Supplementary material for: Carbon emissions from total intravenous vs. volatile anaesthesia for elective cholecystectomy: a pilot observational study
Source: Anaesthesia. 2026 Feb 20;81(6):883–4. doi: 10.1111/anae.70167 (PMC13161729; doi:10.1111/anae.70167)
Supplement: Supplementary file 1 — Table S1. Summary of life cycle assessment methods used to determine CO2e. [file ANAE-81-883-s001.docx]

# Table S1 Summary of life cycle assessment methods used to determine CO_2_e

| **Method employed to determine CO_2_e** | **Number of items** | **Example of item using this method** |
| --- | --- | --- |
| Process-based life cycle assessments | 105 |  |
| Pre-existing process-based life cycle assessments identified and CO_2_e value directly used | 87 | Monocryl 3-0 |
| Pre-existing process-based life cycle assessments identified and further calculations performed using their raw data | 18 | 50 ml syringe |
| Environmentally extended input-output life cycle assessments | 58 |  |
| Hospital invoice data used to determine cost of equipment | 36 | Cannula dressing |
| National public pricing used from NHS Electronic Drug Tariff March 2025 | 11 | Paracetamol 500 mg tablet |
| National public pricing used from British National Formulary edition 89 | 8 | Dexamethasone 3.3 mg ampoule |
| Above methods not suitable, so item excluded from life cycle assessments | 6 | Floseal |
| Hospital invoice data extrapolated to determine cost of equipment | 2 | Single use anaesthetic facemask size 4 (extrapolation involved assuming same cost as size 3 facemask) |
| Public catalogue (Medisave) used to determine cost of equipment | 1 | Temperature probe cover |
| **Total** | **169** |  |
